# Patient-centered primary care for adults at high risk for AUDs: the Choosing Healthier Drinking Options In primary CarE (CHOICE) trial

**DOI:** 10.1186/s13722-017-0080-2

**Published:** 2017-05-17

**Authors:** Katharine A. Bradley, Evette Joy Ludman, Laura J. Chavez, Jennifer F. Bobb, Susan J. Ruedebusch, Carol E. Achtmeyer, Joseph O. Merrill, Andrew J. Saxon, Ryan M. Caldeiro, Diane M. Greenberg, Amy K. Lee, Julie E. Richards, Rachel M. Thomas, Theresa E. Matson, Emily C. Williams, Eric Hawkins, Gwen Lapham, Daniel R. Kivlahan

**Affiliations:** 1 Kaiser Permanente Washington Health Research Institute, 1730 Minor Ave, Suite 1600, Seattle, WA 98101-1466 USA; 2 Center of Excellence in Substance Abuse Treatment and Education (CESATE), Veterans Affairs (VA) Puget Sound Health Care System, Seattle, WA USA; 3 Health Services Research and Development (HSR&D) Seattle Center of Innovation for Veteran-Centered and Value-Driven Care, Seattle, WA USA; 4 Department of Health Services, University of Washington, Seattle, WA USA; 5 Department of Medicine, University of Washington, Seattle, WA USA; 6 Division of Health Services Management and Policy, College of Public Health, The Ohio State University, Columbus, OH USA; 7 Center for Innovation in Pediatric Practice, Nationwide Children’s Hospital, Columbus, OH USA; 8 Department of Psychiatry and Behavioral Sciences, University of Washington, Seattle, WA USA; 9 General Medicine Service, Veterans Affairs (VA) Puget Sound Health Care System, Seattle, WA USA

**Keywords:** Shared decision making, Alcohol use disorder, Care management, Brief interventions, Chronic Care Model, Patient-centered care, Medical management, Veterans, Primary care, Intervention

## Abstract

**Background:**

Most patients with alcohol use disorders (AUDs) never receive alcohol treatment, and experts have recommended management of AUDs in primary care. The Choosing Healthier Drinking Options In primary CarE (CHOICE) trial was a randomized controlled effectiveness trial of a novel intervention for primary care patients at high risk for AUDs. This report describes the conceptual and scientific foundation of the CHOICE model of care, critical elements of the CHOICE trial design consistent with the Template for Intervention Description and Replication (TIDieR), results of recruitment, and baseline characteristics of the enrolled sample.

**Methods:**

The CHOICE intervention is a multi-contact, extended counseling intervention, based on the Chronic Care Model, shared decision-making, motivational interviewing, and evidence-based options for managing AUDs, designed to be practical in primary care. Outpatients who received care at 3 Veterans Affairs primary care sites in the Pacific Northwest and reported frequent heavy drinking (≥4 drinks/day for women; ≥5 for men) were recruited (2011–2014) into a trial in which half of the participants would be offered additional alcohol-related care from a nurse. CHOICE nurses offered 12 months of patient-centered care, including proactive outreach and engagement, repeated brief motivational interventions, monitoring with and without alcohol biomarkers, medications for AUDs, and/or specialty alcohol treatment as appropriate and per patient preference. A CHOICE nurse practitioner was available to prescribe medications for AUDs.

**Results:**

A total of 304 patients consented to participate in the CHOICE trial. Among consenting participants, 90% were men, the mean age was 51 (range 22–75), and most met DSM-IV criteria for alcohol abuse (14%) or dependence (59%). Many participants also screened positive for tobacco use (44%), depression (45%), anxiety disorders (30-41%) and non-tobacco drug use disorders (19%). At baseline, participants had a median AUDIT score of 18 [Interquartile range (IQR) 14–24] and a median readiness to change drinking score of 5 (IQR 2.75–6.25) on a 1–10 Likert scale.

**Conclusion:**

The CHOICE trial tested a patient-centered intervention for AUDs and recruited primary care patients at high risk for AUDs, with a spectrum of severity, co-morbidity, and readiness to change drinking.

*Trial registration* The trial is registered at clinicaltrial.gov (NCT01400581).

**Electronic supplementary material:**

The online version of this article (doi:10.1186/s13722-017-0080-2) contains supplementary material, which is available to authorized users.

## Background

 Over 29% of U.S. adults have had an alcohol use disorder (AUD) at some time in their lives [1, 2]. Most will never seek alcohol treatment, but many will receive health care in primary care settings [1, 2]. Experts have recommended patient-centered approaches to management of AUDs in primary care for over 25 years [3–5], but remarkably little research has addressed how practically to achieve this and whether it improves patient outcomes [6–10].

In 2009, we designed the Considering Healthier drinking Options in primary CarE (CHOICE) intervention to manage AUDs as a chronic condition in primary care patients. The CHOICE model of care is a year-long intervention offered by nurses and based on (1) the Chronic Care Model, (2) principles of patient-centered care [11], and (3) promising approaches to addressing AUDs in primary care [12–23]. We hypothesized that, although most primary care patients with AUDs do not engage in alcohol treatment [2], many would be interested in addressing alcohol use as part of patient-centered care from a registered nurse. We further hypothesized that the resulting increased engagement in AUD care would lead to reductions in drinking and related symptoms, and thereby improve health outcomes. In 2010, the National Institute on Alcohol Abuse and Alcoholism (NIAAA) funded a trial to test the effectiveness of the CHOICE intervention in primary care patients at high risk for AUDs in the Veterans Affairs (VA) health care system.

This report has several purposes. First, it summarizes the conceptual and scientific foundations of the CHOICE model of patient-centered care for AUDs. Second, it describes the design of the CHOICE trial and intervention, including all elements needed for replication of the intervention and comparison to other primary care counseling interventions for AUDs, as advocated by experts [24, 25]. Finally, it reports results of recruitment (completed October 2014) and patient-reported characteristics of the enrolled sample at baseline. Main outcomes of the CHOICE trial were under analysis at the time this report was submitted and will be reported in a follow-up paper.

## Conceptual and scientific foundations of the CHOICE model

Primary care patients with AUDs fall on a spectrum from mild to severe AUDs and from low to high readiness to change. The CHOICE model of care was designed to meet the needs of these diverse patients, building on 20 years of research on patient-centered care for other chronic conditions and on specific interventions for AUDs that have been proven efficacious or shown promise in primary care patients previously, described below.

### The Chronic Care Model and collaborative care

The Chronic Care Model, originally described by Wagner and Von Korff [26, 27], provides a framework for managing chronic conditions in primary care. The model, which seeks to develop informed patients who are actively involved in their own care and recognizes the central role of self-efficacy enhancement, has been proven effective in multiple trials [28].

Collaborative care is a team-based approach based on the Chronic Care Model. In collaborative care, a psychiatrist, psychologist, clinical nurse specialist, or nurse care manager offers: population-based identification, outreach and monitoring; non-judgmental and persistent attempts over time to engage patients in care; evidence-based treatments adapted to the patient’s needs and goals as they change; support for patients in gaining the skills needed to manage their conditions at home and in their communities; objective measurement over time to assess whether patients have met their goals; and collaboration between the care manager and the primary care provider. Collaborative care has proven effective when care managers are located in primary care as well as when they are offsite or via telephone [29–31]. Interdisciplinary review of all patients’ progress, where a collaborative care team repeatedly focuses on how to achieve symptom resolution, is a central component of collaborative care, complementing measurement-based treatment (referred to as “treat to target”) [32]. Trials over the past 20 years have demonstrated the power of collaborative care to improve management and outcomes of mood disorders [33–40] and other chronic mental health and medical conditions [41].

### Shared decision-making

Shared decision-making is the foundation of patient-centered care [11] and a central component of the Chronic Care Model and collaborative care [35, 42, 43]. In shared decision-making, medical providers partner with patients to support patients in making health care choices consistent with their values and priorities. Although shared decision-making is often used to support discrete choices like whether or not to have surgery, it is increasingly recognized to be relevant to chronic disease management [44, 45]. Shared decision-making is especially important when there is no single best treatment—no treatment that is so superior and acceptable that all patients would choose it [11, 46, 47]. Shared decision-making is also critically important for treatments that require patients to be involved in treatment delivery [48]. Therefore, shared decision-making is critical for patient-centered care for patients with AUDs, a condition for which most patients do not want the standard treatment [2], the patient must carry out any care plan him or herself, and there is no one treatment that is generally more effective than others [49–51]. Although shared decision-making has been evaluated in patients seeking inpatient treatment for substance use disorders in Europe and resulted in increased patient autonomy and decreased drug use and psychiatric problems at 3 months [52, 53], it is not routinely incorporated into primary care or specialty treatment for AUDs in the U.S.

There are a number of different frameworks for offering shared decision-making [11, 46, 54], but they all generally include three core elements [11, 46, 54]. First, providers help patients recognize that they have options and a decision to make. Second, providers present patients with information on the efficacy, advantages, and disadvantages of each option, including the option of choosing no treatment (usually combined with watchful waiting or active monitoring). Third, providers explore patients’ values, goals and priorities—the guideposts for their decisions—and support them in choosing the option that is best for them at the present time [55].

### Promising approaches to addressing AUDs in primary care

Both chronic care management and shared decision-making are patient-centered approaches used to engage patients in evidence-based care. Below, we summarize evidence-based and promising approaches to managing AUDs that are practical to offer in primary care.

#### Proactive engagement and repeated brief motivational interventions

Almost 50 years ago, Chafetz showed that engaging patients in non-judgmental care focused on meeting their immediate needs resulted in increased engagement in alcohol treatment [56–58]. Brown showed that proactively engaging primary care patients with AUDs in 3-6 sessions of motivational telephone counseling decreased alcohol consumption at 3 months [59], while other studies have also suggested benefits of repeated brief interventions for patients with AUDs [17, 60]. Motivational Interviewing (MI) skills can be particularly helpful in increasing readiness to change as well as engagement in care for AUDs [9, 61].

#### Repeated brief interventions with biomarker feedback

In the first study of population-based brief interventions for heavy drinking, men with elevated serum gamma glutamyl transferase (GGT), a biomarker of hepatic injury often due to heavy drinking [62, 63], were monitored every 3 months for 2 years and advised to decrease drinking if their GGT was elevated [12]. The intervention was associated with decreased sick absences and hospital days and improved survival [12, 13]. Willenbring demonstrated that a similar intervention was effective in patients hospitalized for alcohol-related medical conditions who had not accepted referral to alcohol treatment. Those offered primary care to monitor their medical conditions with alcohol-related biomarkers (e.g. liver function tests) had increased abstinence, even though patients did not initially have a goal of abstinence [14, 15]. Monitoring amylase in patients with pancreatitis [22] or carbohydrate deficient transferrin (CDT) in patients with diabetes and hypertension [64] has also improved alcohol-related outcomes.

#### Medications for AUDs can help engage patients in care and improve drinking outcomes

Three medications for AUDs have been FDA approved (naltrexone, acamprosate and disulfiram) [23, 65, 66]. The COMBINE trial showed that naltrexone was effective with Medical Management, without specialty addictions care. Medical Management in the COMBINE trial was similar to care management of diabetes, heart failure, or depression by nurses in primary care [21]. It included review of medication adherence, side effects, and support for self-management with encouragement to attend supports for sobriety such as Alcoholics Anonymous (AA) [21]. Moreover, in COMBINE, Medical Management with placebo was more effective on some outcomes than state-of-the-art professional addictions counseling alone [21, 67]. Therefore, primary care patients taking medications for AUDs with Medical Management might benefit from both medication and placebo effects. Recently, Oslin and colleagues showed that proactive engagement with MI skills and treatment with naltrexone increased engagement and decreased heavy drinking compared to referral to alcohol treatment for primary care patients with alcohol dependence [6].

## Methods

### Design of the CHOICE trial

The CHOICE intervention was designed to meet the needs of primary care patients irrespective of the severity of their AUDs or readiness to change. The intervention was tested in a randomized controlled trial from 2011 to 2015. This section describes the design of the trial, followed by a detailed description of the CHOICE intervention.

#### Study setting

CHOICE was conducted at three sites of the VA Puget Sound Health Care System. The VA was chosen as the setting for the trial because it conducts annual alcohol screening allowing identification of patients at increased risk of AUDs, typically under-diagnosed in medical settings [68, 69]. In addition, population-based alcohol screening in the VA would allow the CHOICE trial to compare patients who were enrolled into the trial, to those who were available to be screened and recruited but were not enrolled. The VA’s population-based alcohol screening also allows evaluation of drinking outcomes after the formal 12 month follow-up ends. The VA also ensured access to specialty alcohol treatment for patients who were interested.

#### Eligibility criteria

Primary care patients from the three study sites were eligible if they were 21–75 years old and at high risk for AUDs based on self-report of frequent heavy drinking on a telephone screen. Table 1 lists inclusion and exclusion criteria. Patients less than 21 are rarely seen in the VA, and patients over 75 years were excluded due to limited information on the safety of AUD medications in the elderly. Patients who reported 4 or more drinks in a day for women and 5 or more drinks for men [70] at least twice weekly, or once a week for patients with prior alcohol treatment [71], were eligible (Table 1). Although the intervention was designed for patients with AUDs, many patients with AUDs might under-report symptoms (especially those not interested in changing); therefore, having a DSM-IV AUD diagnosis was not an eligibility criterion. Exclusions were limited to recent engagement in alcohol treatment (past 90 days), so as not to interfere with ongoing care, those required for safety (e.g. pregnancy), and those that would decrease likelihood that participants would be lost to follow-up (Table 1).

**Table 1:** Inclusion and exclusion criteria for the CHOICE trial

	Assessed via…				
	EHR	Primary care provider review	Manual EHR review	Telephone screen	Baseline in-person visit
Inclusion criteria					
Receiving primary care in study clinic	X		X	X^a^	X^a^
Age 21–75 years	X		X	X^a^	X^a^
Frequent heavy drinking^b^				X	X
Exclusion criteria					
Medically or psychiatrically unstable		X	X		X
Cognitive impairment		X	X	X	X
Less than 1 year life expectancy		X	X		
Alcohol treatment in past 90 days	X		X	X	X
Current pregnancy or planned next year			X	X	X
Enrolled in current VA trial			X		
Behavioral warning flag in EHR	X		X		
Leaving VA facility in next year				X	X
VA employee	X		X	X^a^	X^a^
Excluded by primary care provider		X			
No valid phone or address	X		X		

^a^Only for self-referred patients

^b^Self report of 4 or more drinks (women) and 5 or more (men) at least twice a week, or at least once a week for patients who reported previous alcohol treatment

#### CHOICE trial recruitment procedures

**Fig. 1:**
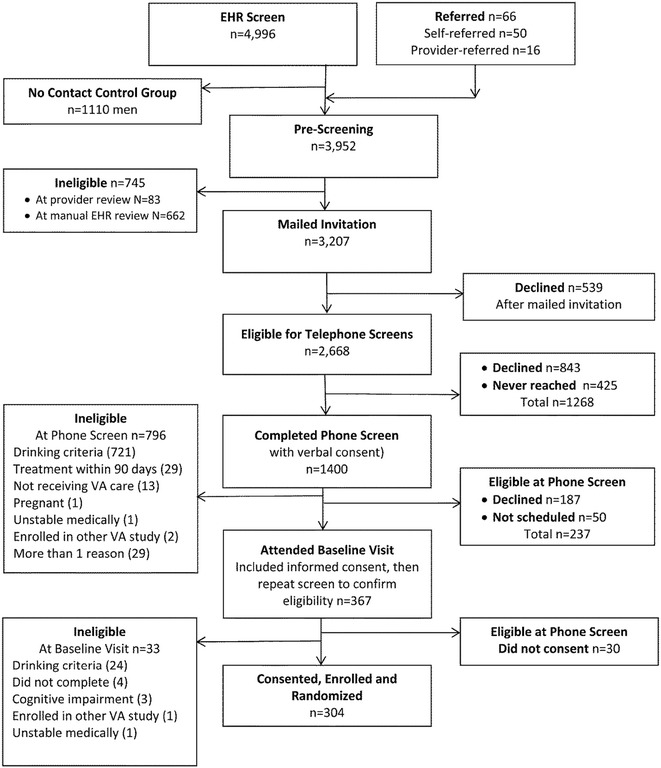
Patients identified for recruitment from the EHR: ineligible, declining, never reached, and enrolled

CHOICE recruitment required a number of steps to protect privacy and safety (Fig. 1).

##### Pre-screening

Review of the electronic health record (EHR) identified patients eligible for screening and possible recruitment because they received primary care at one of the three study sites and had screened positive for unhealthy alcohol use on the AUDIT-C at ≥4 points for women and ≥5 points for men [72]. In addition, providers were allowed to refer patients to the study, and flyers were posted to allow patients to self-refer. Before being sent an invitation letter for CHOICE study screening, patients identified from the EHR were reviewed for appropriateness for the trial by their primary care providers, followed by manual EHR review by study staff for exclusions (Table 1).

The CHOICE trial included a randomly selected “No Contact Control Group” to allow evaluation of the possible effects of recruitment and assessment alone (assessment reactivity) [73–77]. About 25% of men who were otherwise eligible for recruitment based on EHR prescreening were never contacted by the study. Women were not included in the No Contact Control Group to maximize the number of women in the trial because women make up less than 10% of VA patients.

##### Mailed invitation

Patients were sent a letter inviting them to be screened for a study testing “a program designed to improve the health of Veterans who drink alcohol” (Additional file 1: A—Recruitment Materials). The invitation was either signed by their primary care provider or the principal investigator if providers preferred. The letter included a $2 pre-incentive [78–80] and allowed patients to opt out of being called for study screening by returning a post card or calling a toll free number.

##### Telephone screening

Study coordinators telephoned patients for screening and possible recruitment (after verbal consent), if patients had not declined after mailed invitations. Women were preferentially screened in order to maximize their recruitment. Eligible patients, based on the telephone screen [81–85], were invited to schedule an in-person visit with an enrollment coordinator to determine interest in participating, obtain written informed consent, determine final eligibility, and administer baseline assessments (Additional file 1: A—Recruitment Materials).

##### Baseline enrollment visit

Three enrollment coordinators conducted baseline assessments. At the baseline visit, informed consent was obtained and patient eligibility was established including repeat assessment of drinking eligibility criteria and cognitive assessment with the Mini-Cog (Table 1; Additional file 1: A—Recruitment Materials) [81–85]. Eligible participants completed all baseline assessments in person (Table 2), including laboratory tests, and self-administered screens for unhealthy alcohol use (past year), depression (past 2 weeks), and past month posttraumatic stress disorder (PTSD), using the Alcohol Use Disorders Identification Test (AUDIT) [86], the 9-item Patient Health Questionnaire (PHQ-9) [87], questions about experiences of trauma [88], and the PTSD Checklist (PCL) [89], respectively. Alcohol and drug use disorders in the past year were assessed with the Mini International Neuropsychiatric Interview (MINI). Alcohol consumption was assessed with a 28-day time-line follow-back (TLFB) [90], and alcohol-related problems ever and in the past 3 months were assessed with the Short Inventory of Problems (SIP) [91, 92]. Patients were compensated $10 for rescreening at baseline and another $10 for the baseline assessment, which lasted ~1 h.

**Table 2:** Assessments and laboratory tests at baseline,  3 and 12 months

	Timeframe	Baseline	3 months	12 months
Telephone screen				
Number of heavy drinking days in the past 28 days	Past 28 days	X^a^		
Prior treatment	LT, P90D	X^a^		
Self-administered surveys				
Alcohol Use Disorder Identification Test (AUDIT) [86]	PY	X^a^		X
AUDIT consumption questions (AUDIT-C)	Past 4 weeks			X
9-item Patient Health Questionnaire (PHQ-9) [87]	Past 2 weeks	X^a^		X
Traumatic experience ever (MINI) [88]	LT, PM	X^a^		
PTSD Check List (PCL) [89]	PM	X^a^		
Interview				
Mini-Cog [81–83]	Current	X		
Health habits	PM, current	X	X	X
12-item Short Form (SF-12) Veterans Version [134, 135],	PM	X	X	X
28 day Time Line Follow Back Interview, alcohol [90]	Past 28 days	X	X	X
Short Inventory of Problems (SIP) [91, 92]	LT, P3Mo	X	X	X
DSM-IV Major Depressive (current, past) (MINI) [88]	LT, P2W	X^a^		
DSM-IV Panic disorder (current, lifetime) (MINI) [88]	LT, PM	X^a^		
Generalized Anxiety Disorder Screen (GAD-7) [115]	P2W	X^a^		
DSM-IV AUD (MINI) [88]	PY	X^a^		X
Craving question [116] for DSM-5 AUD [136, 137]	LT	X^a^		X
DSM-IV DUD (MINI) [88]	LT, PY	X^a^		
DUD craving question	LT	X^a^		
Importance, readiness, confidence rulers [138, 139]	None	X	X	X
Important People Inventory [140]	P4Mo	X		
Socio-demographics	Current	X		X
Family history AUDs (parents, sibling children)	LT	X		
Alcohol treatment or related services (e.g. AA) [1]	LT, P3Mo, PY	X		X
AA meetings [141]	LT, PY			X
Distance to appointments	None	X		
Non-VA emergency department visits	P3Mo, PY	X	X	X
Non-VA hospitalizations	P3Mo, PY	X	X	X
Other non-VA care	P3Mo, PY	X	X	X
Laboratory tests				
Gamma glutamyl transferase (GGT) [117]	None	X^a^		X
Carbohydrate deficient transferrin (CDT) [117]	None	X^a^		X
Mean corpuscular (MCV) [117]	None	X^a^		X

^a^Available to CHOICE RNs at Baseline; *PM* past month, *LT* lifetime, *PY* past year, *P90D* past 90 days, *P3Mo* past 3 months, *P4Mo* past 4 months, *P2W* past 2 weeks

#### Randomization

After baseline assessments, participants were randomized (1:1) to either the CHOICE Intervention or Usual Care control. Randomization was stratified on sex, DSM-IV alcohol dependence (yes/no), and site (n = 3) in permuted blocks of varying size. Randomization was conducted using computer-generated random numbers, and allocation was concealed by computer software.

#### Usual alcohol-related care in the study primary care clinics

Prior to the start of the CHOICE trial, all study sites had implemented annual alcohol screening and a single brief intervention for those who screened positive at AUDIT-C scores ≥ 5 points, as well as integrated mental health care, as part of a national requirement [93–96]. At VA Puget Sound, this included use of collaborative care for depression, but not AUDs, as well as co-located psychiatrists and psychologists. Beginning in 2010, the study sites also implemented patient-centered medical homes, referred to as Patient Aligned Care Teams (PACTs) in the VA. The PACTs had increased resources for nurse care management [97] but did not provide care management for AUDs. As a result, the standard management of AUDs in primary care was still referral to specialty addiction treatment during this trial.

#### Data collection

The CHOICE trial measured patient-reported outcomes, laboratory biomarkers, and VA and non-VA health care utilization (including care by the CHOICE team) at 3 and 12 months (Table 2). Main outcome measures at 12 months—percent heavy drinking days in the past 28 days [98] and a “good drinking outcome” (drinking below NIAAA recommended limits in the past 28 days without alcohol-related symptoms in the past 3 months on the SIP) [21, 99]—were collected by the Kaiser Permanente Washington Health Research Institute Survey Research Department (an independent survey team) using computer assisted telephone interviews with interviewers blinded to treatment assignment. Telephone interviews were used to provide more flexibility than in-person interviews. Computers, tablets, or smartphones were not used because many patients did not own them when the study was designed (2009–2010), and using a common mode of assessment was preferred.

At 12 months, 4 study coordinators arranged for participants to complete additional self-report questionnaires (e.g. AUDIT and PHQ-9) and laboratory tests. However, study coordinators could not be reliably blinded to treatment assignment due to their interactions with patients.

#### The CHOICE intervention

The 12-month CHOICE intervention consisted of chronic care management provided by one of two registered nurses (“CHOICE nurses”) and supervised by an interdisciplinary team (“CHOICE team”). A decision was made not to ask participants’ primary care providers to prescribe AUD medications—unlike collaborative care for depression—after initial discussions with primary care clinicians indicated many would not want to prescribe AUD medications. A CHOICE nurse practitioner, a VA primary care provider, was available to prescribe and manage medications for AUDs. The CHOICE nurses had prior research experience as care managers for diabetes, heart failure, and/or depression [100, 101]. However, neither the nurses nor the nurse practitioner had prior experience managing AUDs or medications for AUDs.

##### CHOICE nurse and nurse practitioner training

The first nurse and nurse practitioner were initially given articles and protocols to read [14, 21, 33, 36, 43, 65, 102–108], then received 16 h of training, which included motivational interviewing (MI) skills focused on engagement, ways to increase the importance patients placed on changing their drinking, and building patient self-efficacy. An additional 2 h of training addressed medications and laboratory monitoring [21, 65]. The second nurse was added to the study after the caseload of the first increased. The second nurse had experience with MI skills from working in a prior trial [100] and required fewer hours of training (6 total). Nurses participated in a site visit to the VA specialty addiction treatment program, which they found invaluable for informing patients about available services.

##### Proactive telephone outreach

A nurse telephoned each intervention group patient as soon as possible after randomization to invite him/her to an initial visit (“engagement visit”) [109]. As above, patients had been told that randomly selected patients would be offered services, but they were not required to accept them, making CHOICE an “encouragement trial” [110–113]. Nurses made every effort to schedule the initial engagement visit in person. At two of the three sites, nurse office visits were not in the primary care clinics due to space constraints and were instead in research offices nearby at the same site.

##### Review of the medical record and baseline assessments

Prior to the engagement visit, nurses reviewed patients’ EHRs for all medical and mental health conditions potentially impacted by alcohol use and any alcohol treatment history. They also reviewed screens and brief instruments administered during the CHOICE baseline assessment (Table 2) [86–89, 114–116] and alcohol biomarker results: GGT, CDT, and red cell mean corpuscular volume (MCV) [117, 118].

##### Patient engagement

The goal of the engagement visit was to get to know the patient—his/her goals, values, and life priorities—and to engage the patient in a caring, non-judgmental relationship that could support him/her in making changes [119]. The CHOICE nurses learned about relationships and activities important to the patient and how those activities related to the patient’s drinking. Engagement visits typically lasted ~60–90 min, and, if more time was needed, engagement was sometimes spread over two encounters. The nurse used a template to document the engagement visit in the EHR, with the primary care provider copied on the note, which was also provided to the CHOICE team (Additional file 1: B—Fictional Example of Template for EHR Engagement Note).

##### Repeated brief interventions using MI skills and shared decision-making

After engagement, usually at the second visit, CHOICE nurses provided feedback from patients’ baseline assessments. Abnormal lab tests and medication options for AUDs were reviewed irrespective of whether or not patients met criteria for DSM-IV AUDs on the CHOICE baseline assessment in an effort to link drinking and health and decrease stigma. Per patient interest, nurses also provided handouts about the medical effects of drinking (such as calories from alcohol, a topic of interest to many patients) to enhance motivation for change [108, 120]. At the same time, nurses made it clear to patients that any decision to change their drinking was up to them and that the nurse would continue to support them irrespective of their individual goal or readiness to change. Patients who were not interested in changing were invited to consider self-monitoring (documenting their drinking daily in a manner convenient to them). Patients interested in making changes were supported in considering whether they wanted to cut down or stop drinking. Information was also provided on the range of treatment options that could support patients in making changes in their drinking, including biomarker monitoring and feedback, CHOICE nurse visits, AUD medication, specialty alcohol treatment, and AA, or other mutual help.

##### Ongoing patient support and proactive monitoring

Over the 12 month intervention, nurses continued to offer ongoing support, monitoring, MI, and shared decision-making if patients were interested in considering change(s). Follow-up visits were in person or via telephone, depending on patient preference, and lasted from 5 to 60 min, depending on patient needs and preference. Nurses and patients collaboratively determined visit frequency; generally visits tapered from weekly to monthly. Nurses called patients who missed appointments and continued follow-up with patients even if patients did not respond, leaving voice messages or sending letters at least once a month (unless patients requested otherwise). The content of follow-up visits varied depending on patients’ needs, but visits always included efforts to build motivation and self-efficacy. Patients’ progress was monitored in several ways during these visits per patient preference: self-monitoring with a diary or other calendar, use of laboratory biomarkers, and/or the AUDIT. At each visit, nurses and patients also assessed progress on their goal(s), problem-solved challenges to goal attainment, and considered additional treatment services as appropriate. Nurses helped coordinate access to the patient’s chosen treatment, including inpatient alcohol withdrawal, if necessary.

##### Weekly CHOICE team meetings

The CHOICE nurses, nurse practitioner, and interdisciplinary CHOICE team—including 2 psychologists, 2 addiction psychiatrists, and 2 primary care internists—met weekly. These meetings were a critical part of the intervention, lasted an hour, and were held in person with the nurses and 3 members of the interdisciplinary team, while the other 3 members were usually on the phone. The CHOICE team was large to allow adequate coverage for weekly meetings for 3.5 years. Team meetings used a roster of intervention patients (Additional file 1: C—Fictional CHOICE Patient Roster) that included: age, sex, randomization date, number of visits to date, last visit, last 2 values for each laboratory biomarker (CDT, MCV, GGT), most recent AUDIT and PHQ-9 scores, last AUD medication and date, and comments (e.g. enrolled in treatment). Each week, nurses presented new patients and took turns reviewing their entire roster, discussing about 25–30 patients in an hour with a focus on those patients who were not engaged or not making progress. The CHOICE nurse practitioner also consulted about medications during CHOICE team meetings. In addition to the larger CHOICE team meeting, the nurses also met with one psychologist and internist for an hour most weeks to discuss new and challenging patients and to review MI skills. Discussions with both the large and small groups were patient-focused and aimed to support the nurses in developing persistent caring relationships and adapting care when patients did not respond. There was no systematic fidelity monitoring of nurse adherence to the treatment model.

##### Communication with primary care providers and other VA providers

**Fig. 2:**
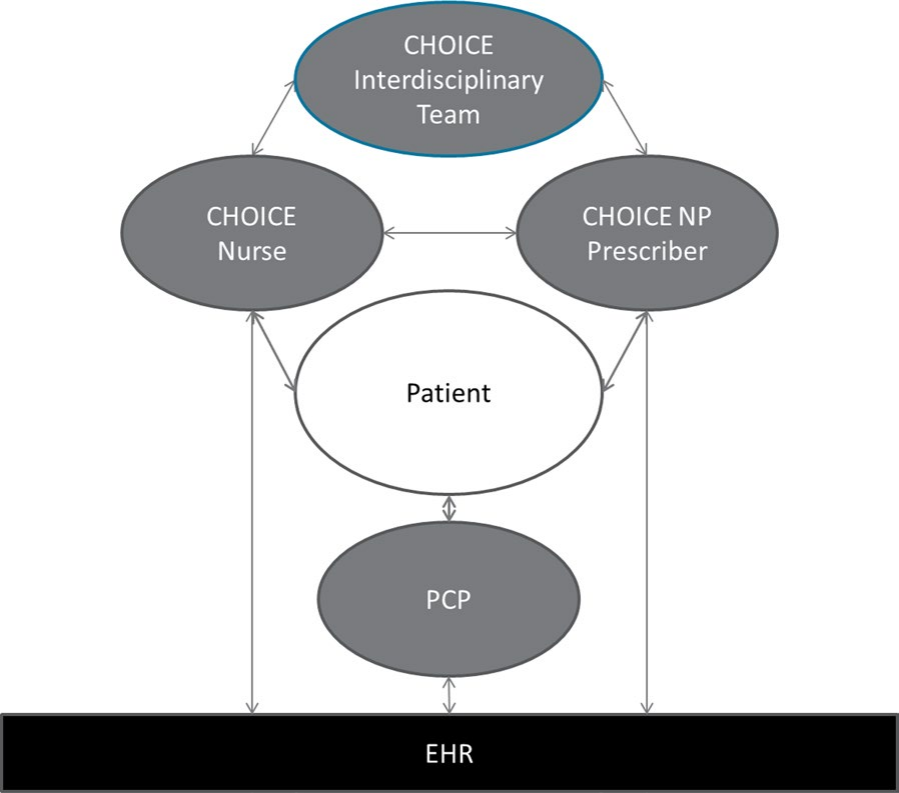
Diagram of the CHOICE intervention team. *EHR* electronic health record; *PCP* primary care provider

The CHOICE intervention did not require VA primary care providers to be actively involved in their patients’ alcohol-related care (Fig. 2). However, nurses documented all visits in the EHR and communicated with the primary care provider via notes in the EHR by copying the primary care provider or asking them to co-sign the note when medically relevant information was provided (e.g. a need for depression treatment, medically-managed alcohol withdrawal, or plans for AUD medications).

## Results

### Recruitment and characteristics of CHOICE participants

#### Screening and recruitment

Figure 1 depicts the recruited patient sample and numbers of patients who were: found ineligible; not willing to be screened or participate; never reached; or eventually enrolled. EHR screening initially identified 4996 primary care patients between the ages of 21 and 75 who screened positive for unhealthy alcohol use. After 1110 men were randomized to the No Contact Control Group, and primary care providers referred another 16 patients, and 50 patients self-referred, a total of 3952 were available for screening and recruitment (3429 men and 523 women). A total of 1574 were found ineligible based on provider review (83), manual chart review (662), telephone screening (796), or baseline interviews (33). Of the remaining 2378 patients not known to be ineligible, 425 (18%) were never reached for telephone screening, 1649 (69%) declined to participate, and 304 (13%) were enrolled in the trial. Of the 1649 patients who declined to participate, 1382 (84%) did so before eligibility was determined (539 after mailed invitations and 843 when initially reached for telephone screening); only 267 (16%) declined participation after they were preliminarily deemed eligible based on telephone screening (187 explicitly declining participation after screening, 50 never scheduling, and 30 not consenting after coming for in-person visits).

Of 1400 patients who completed telephone screens, 829 (59%) were found ineligible at telephone screening (796), or at baseline in-person assessments (33). As shown in Fig. 1, most ineligible patients were ineligible because they did not drink at levels required by the study (87%). Of the remaining 571 patients who were potentially eligible based on phone screens, 47% declined (Fig. 1), and a total of 304 patients (53%) enrolled in the trial, including 275 men (90.5%) and 29 women (9.5%).

#### Differences between enrolled and non-enrolled patients

Age, gender, and AUDIT-C scores from the VA’s EHR were used to compare the 304 patients enrolled in the CHOICE trial to: (1) the 2074 patients who declined to participate (1649) or were never reached (425); and (2) the 1574 patients found ineligible at screening. Compared to the 2074 patients who declined to participate or were never reached, the 304 patients who enrolled in the trial were of similar age (mean age 51.4 years enrolled vs. 51.0 years declined/not reached; Wilcoxon ranked sum test *p* = 0.89) and gender (90.5% male in the enrolled sample vs. 87.8% in the declined/not reached sample; Fisher exact test *p* = 0.19). However, enrolled patients had higher mean AUDIT-C scores (7.0 points in the enrolled sample vs. 6.4 points in the declined/not reached sample; *p* < 0.0001). The difference in AUDIT-C scores was statistically significant for both men (7.2 vs 6.6; *p* < 0.0001) and women (5.5 vs 4.8; *p* 0.035). Compared to the 1574 patients determined ineligible, the 304 patients who enrolled in the trial were older (51.4 vs 47.8 years; *p* = 0.0003) with age differences among men (52.2 vs 48.7; *p* = 0.001) but not women (43.7 vs 42.8; *p* = 0.68), and enrolled patients were more likely to be male (90.5 vs 84.8%; *p* = 0.009) and had higher AUDIT-C scores (7.0 vs 6.4; *p* < 0.0001), using the same approaches for statistical testing as for above comparisons to the 2070 declined/not reached. Because, some referred patients lacked these EHR measures, especially AUDIT-C screens—missing for up to 20 of enrolled, 14 of declined/not reached, and 32 of ineligible patients—analyses were repeated excluding referred patients, and results were not meaningfully changed (Additional file 1: Characteristics of Patients Enrolled, Ineligible and Declined/Not Reached).

#### Enrolled sample

The 304 patients who enrolled in the trial were predominantly white, with ages ranging from 22 to 75 years (mean 51.4); 81.3% were married at some time in their lives (Table 3). Tobacco smoking (44.1%), depression (45.4%), anxiety (30.3%), and PTSD (41.2%) were relatively common in the 304 participants in the trial, and almost 1 in 5 met criteria for non-tobacco, non-alcohol DSM-IV drug use disorders (DUDs; 18.8%). Participants reflected a spectrum of unhealthy alcohol use with a median full AUDIT score of 18 [interquartile range (IQR) 14–24], and 51% reported drinking 6 or more drinks daily or almost daily on the AUDIT (Table 3). At baseline, the median percent days of heavy drinking was 64% (IQR 35–93%), and the median past 3 month SIP score was 7 (IQR 2–13). Overall, 73.4% of participants met DSM-IV criteria for past year AUD (59.2% for dependence alone) based on the MINI, and over half reported having “gone anywhere or seen anyone for a reason related in any way to your drinking (physician, counselor, AA, other community agency or profession)” [1]. Although 71.4% reported that it was “somewhat” to “very” important to change their drinking, only 44.1% were very confident they could change. The self-reported readiness to change drinking at baseline varied (Table 3), with a median readiness score of 5 (IQR 2.75–6.25) on a 10-point Likert scale.

**Table 3:** Baseline characteristics of study sample (N = 304)

	N	%
Men	275	(90.5)
Age (years)		
21–35	54	(17.8)
35–49	65	(21.4)
50–64	131	(43.1)
65–75	54	(17.8)
Race/ethnicity		
Black	39	(12.8)
White	206	(67.8)
Native American	25	(8.2)
Native Hawaiian/PI	5	(1.6)
Bi/multiracial	22	(7.2)
Asian	2	(0.7)
Other	5	(1.6)
Hispanic	21	(6.9)
Marital status^a^		
Never married	56	(18.4)
Married/partnered	136	(44.7)
Separated	13	(4.3)
Divorced	91	(29.9)
Widowed	7	(2.3)
Education		
High school/GED or less	65	(21.4)
Some college/tech school	170	(55.9)
College or post graduate	69	(22.7)
Income^a^		
<$15,000	49	(16.1)
$15,000–29,999	62	(20.4)
$30,000–59,999	96	(31.6)
$60,000–89,999	57	(18.8)
≥$90,000	38	(12.5)
Behavioral health screening		
Smokes tobacco (some/daily)	134	(44.1)
Depression (PHQ-9 ≥10 points)	138	(45.4)
Suicidal thoughts >half days (PHQ-9)	16	(5.3)
GAD (GAD-7 ≥10 points)	92	(30.3)
DSM-IV PTSD based on PCL^a^	125	(41.2)^a^
DSM-IV Non tobacco, DUD, past year (MINI)	57	(18.8)
Alcohol use, problems and history		
Drinks ≥6 drinks ~daily (AUDIT Q#3)	155	(51.0)
AUDIT score ≥20	134	(44.1)
DSM-IV AUD, past year (MINI)	223	(73.4)
DSM-IV alcohol dependence, past year (MINI)	180	(59.2)
Prior alcohol treatment or related services	169	(55.6)
Patient reported importance of change		
Not important	87	(28.6)
Somewhat important	110	(36.2)
Very important	107	(35.2)
Patient reported confidence in ability to change		
Not confident	58	(19.1)
Somewhat confident	112	(36.8)
Very confident	134	(44.1)
Patient reported readiness to change		
Not ready	98	(32.2)
Somewhat ready	130	(42.8)
Ready	76	(25.0)

^a^Percentages do not sum to 100% due to missing values; PCL had 4 missing

## Discussion

The purpose of this report was to provide a rationale and description of the CHOICE intervention and trial as recommended by experts [24]. The CHOICE intervention was designed to improve outcomes of primary care patients at high risk for AUDs by offering patient-centered chronic care management using MI skills and shared decision-making over 12 months. Patients were recruited and engaged in the intervention regardless of their recognition of problems due to drinking, severity of unhealthy alcohol use, or readiness to change drinking. The CHOICE trial will evaluate the effectiveness of the nurse care management intervention for resolving high risk drinking and alcohol-related symptoms. Baseline data show that the trial recruited a sample of patients with varying severity of unhealthy alcohol use, readiness to change, and a significant burden of mental health and other substance use conditions.

Randomized controlled trials of interventions to manage AUDs in primary care patients have had varying designs and results [6, 7, 9, 14, 59]. Understanding the implications of their varying results requires a detailed knowledge of the interventions, populations studied, and trial designs. Unfortunately, all too often, neither is adequately characterized in relatively brief reports in medical journals [24]. As a result, experts have recommended several frameworks to improve reporting and interpretation of the literature on behavioral interventions that can be offered in primary care [24, 25, 121, 122]. One recommendation is that a checklist, the Template for Intervention Description and Replication (TIDieR), be used to ensure adequate description of behavioral interventions and the design of trials conducted in primary care [25]. This report includes those TIDieR elements—intervention components, materials used in the intervention, procedures and processes used, intervention providers and their expertise, modes of delivery, locations, whether the intervention was personalized or adapted, and how adherence was assessed—that are known before blinded analyses of main and secondary outcomes are completed.

Randomized controlled trials of behavioral interventions in primary care samples fall on a continuum from purely “explanatory,” efficacy trials to more “pragmatic” effectiveness trials, and different dimensions of a trial can vary in the extent to which they are explanatory or pragmatic [123]. The CHOICE trial is considered a blended pragmatic effectiveness-efficacy trial based on the 10 indicators of the “Pragmatic-Explanatory Continuum Indicator Summary (PRECIS) [123]. Pragmatic elements of the CHOICE trial include: non-stringent criteria for inclusion/exclusion, the intervention which was flexible and not monitored systematically for fidelity, and use of telephone interviews for outcomes, all designed to maximize flexibility and thereby external validity. Exclusion criteria were limited, and the primary inclusion criterion relied on the frequency of heavy drinking. Frequent heavy drinking was used as an inclusion criterion because it is strongly associated with AUDs [70, 124] commonly assessed by brief alcohol screens in primary care [125], and because AUDs are often under-recognized in primary care [69], and patients under-report AUD symptoms due to stigma [126]. Consistent with shared decision-making principles, the CHOICE intervention was adapted to patient preferences regarding drinking goals, frequency of contacts, setting (telephone vs in person), and treatment options. The comparison condition was usual primary care with no additions. Measurements were pragmatic in that main outcomes were measured by telephone at diverse times of day, seven days a week, to increase flexibility and acceptability to patients. Only limited secondary outcomes were collected from patients at three months, with EHR data used to measure other secondary outcomes to decrease participant burden and assessment reactivity. Finally, embedding the trial in the VA health care system enabled future use of annual alcohol screening and the EHR to evaluate longer term outcomes after the main patient reported outcomes at 12 months. While these pragmatic elements of the trial were expected to limit its explanatory potential, especially if the intervention was not effective, explanatory elements below were designed to counter those limitations.

Despite the above pragmatic elements of the CHOICE trial design, other elements were less pragmatic. The recruitment procedures included at least seven interactions between research staff and all patients (including the control group) before the 12 month assessment: an invitation letter; a telephone screen; an appointment reminder letter with consent form; an in-person baseline assessment; a letter with results of randomization; a 3-month reminder letter; and a telephone assessment at 3 months, with each contact often requiring many attempts to reach or schedule the patient. These could act as a barrier to participation, but also as a catalyst to change by increasing self-awareness of alcohol-related problems in both groups [76, 77, 122, 127]. The 13-page written informed consent document, patient-level randomization, and measurement of main outcomes with patient interviews were also on the explanatory end of the continuum. The randomly selected No Contact Control Group of men will allow evaluation of the potential impact of recruitment and assessment at baseline and three months, allowing explanatory analyses of changes in drinking in the usual care group—albeit with pragmatic measures. Elements of the intervention, which were also less pragmatic, included having CHOICE nurses manage only AUDs while referring other medical or mental health issues to primary care teams, not requiring that primary care providers provide any of the alcohol-related care including prescription of AUD medications, location of the nurses outside primary care clinics, and collaboration between CHOICE RNs and primary care providers limited largely to medications or medical issues.

The CHOICE trial included 304 primary care patients, a low proportion of those who were initially identified from the EHR for screening and recruitment for the trial. Of the 3952 patients initially identified, 1807 (46%) either declined before eligibility was determined (1382) or were never reached (425). Of the 571 patients potentially eligible based on phone screening for the trial, 53% enrolled. Most patients who were ineligible did not drink above levels required for inclusion. Given all the demands of screening, recruitment, and measurement above, the low enrollment rate among all VA patients eligible for screening and recruitment was as expected and similar to the proportion of patients who engaged in prior effectiveness-efficacy trials of collaborative care [100]. CHOICE was not designed to maximize reach of the intervention unlike many pragmatic trials [128–131]. At the same time, our analyses show that the 304 enrolled patients were similar in age and gender to the 2074 who declined or were never reached but had higher mean AUDIT-C scores indicative of more severe unhealthy alcohol use [132]. Given the positive association between severity of unhealthy alcohol use and readiness to change [133], we suspect recruited patients also had greater readiness to change than those who were not recruited. Despite efforts to oversample women, the CHOICE trial did not enroll adequate numbers of women to generalize trial findings to women.

Two prior approaches to chronic care management of AUDs in primary care, tested in recent randomized controlled trials, had markedly differing outcomes from each other: one showing a benefit of chronic care management and the other finding no benefit [6, 7]. The two trials both blended pragmatic elements (intended to increase generalizability) with explanatory elements (intended to improve internal validity) but the samples, interventions, and comparisons in those studies differed in important ways from the CHOICE trial. A trial in the VA conducted by Oslin et al. [6] recruited primary care patients who met criteria for DSM-IV alcohol dependence irrespective of their readiness to change (excluding patients with other substance use and psychotic disorders). In contrast, the AHEAD trial by Saitz and colleagues included patients with DSM-IV AUD or other substance use disorders recruited outside primary care without exclusions, and many were seeking treatment. The median readiness to change was 10 (on a scale of 1–10) [7]. CHOICE, in contrast, recruited primary care patients with frequent heavy drinking, irrespective of comorbidity or symptoms of AUDs, and although most patients met criteria for AUDs, the median readiness to change was 5. The intervention in the Oslin trial was delivered by behavioral health specialists (nurse or psychologist) [6], and the intervention in AHEAD was delivered by an interdisciplinary team (nurse, internist, social worker and psychiatrist) [7], while the CHOICE intervention was delivered by non-specialist nurses, supported weekly by an interdisciplinary team. The comparators of the three studies also differed; the Oslin study compared the intervention to referral to treatment [6], whereas AHEAD compared the intervention to referral to specialty addictions treatment and augmented primary care [7]. In contrast, the comparator in the CHOICE trial was usual primary care within a health care system that provided access to the full continuum of addiction specialty care. When the CHOICE trial results are available, thorough examination of the differences in the three studies may help identify promising elements of patient-centered care management of AUDs.

## Conclusion

 The CHOICE trial tested a novel intervention of 12 months of patient-centered care—offering repeated brief interventions using MI skills and shared decision-making—to engage patients at high risk for AUDs in alcohol-related care irrespective of their recognition of symptoms due to drinking, the presence or severity of AUDs, and/or readiness to change. Results of recruitment reported here suggest that the trial enrolled patients with the full spectrum of AUDs, of varying severity and readiness to change. Future reports will present findings regarding engagement with the intervention and main outcomes: reduced heavy drinking and drinking below recommended limits without symptoms.

## Additional file

**Additional file 1.** Documentation regarding study procedures including recruitment materials (A), fictional example of initial CHOICE progress note template (B), fictional CHOICE patient roster (C), and differences between enrolled and non-enrolled samples.

## Abbreviations

AA: Alcoholics Anonymous
AUD: alcohol use disorder
AUDIT: Alcohol Use Disorder Identification Test
CDT: carbohydrate-deficient transferring
CHOICE: Choosing Healthier Drinking Options in primary CarE study
COMBINE: The Combined Pharmacotherapies and Behavioral Interventions study
DSM: Diagnostic and Statistical Manual of Mental Disorders
DUD: non-alcohol, non-tobacco drug use disorders
EHR: electronic health record
FDA: Food and Drug Administration
GAD-7: 7-Item Generalized Anxiety Disorder screen
GGT: gamma glutamyl transferase
MCV: mean corpuscular volume
MI: motivational interviewing
MINI: Mini International Neuropsychiatric Interview
NIAAA: National Institute on Alcohol Abuse and Alcoholism
PHQ-9: 9-item Patient Health Questionnaire
PTSD: Post-traumatic stress disorder
SF-12: 12-item Short Form
SIP: Short Inventory of Problems
TLFB: time-line follow-back
VA: Veterans Affairs
