# Supplementary material for: Patient-centered primary care for adults at high risk for AUDs: the Choosing Healthier Drinking Options In primary CarE (CHOICE) trial
Source: Addict Sci Clin Pract. 2017 May 17;12:15. doi: 10.1186/s13722-017-0080-2 (PMC5436432; doi:10.1186/s13722-017-0080-2)
Supplement: Supplementary file 1 — Additional file 1. Documentation regarding study procedures including recruitment materials (A), fictional example of initial CHOICE progress note template (B), fictional CHOICE patient roster (C), and differences between enrolled and non-enrolled samples. [file 13722_2017_80_MOESM1_ESM.pdf]

Supplements

Table of Contents

A. **RECRUITMENT MATERIALS** 2

1. Invitation Letter 3
2. Information Sheet 4
3. Brief Telephone Screen Script 5
4. Consent Form 10

B. **FICTIONAL EXAMPLE OF TEMPLATE FOR EHR ENGAGEMENT NOTE** 23

C. **FICTIONAL CHOICE PATIENT ROSTER** 25

D. **COMPARISON OF ENROLLED AND NON-ENROLLED SAMPLES** 27

SUPPLEMENT A: Recruitment Materials

DEPARTMENT OF VETERANS AFFAIRS
VA Puget Sound Health Care System
1660 S. Columbian Way
S-152-HSRD
Seattle, WA 98108

CHOICE A Research Study for Veterans

< Patient Name > < Date >
< Patient Address >
< Patient City, State Zip >

Dear <Patient's Name>,

I am writing you to let you know about the "CHOICE" study— a research study for Veterans who get care at VA Puget Sound primary care clinics. The study is looking for volunteers, and I thought you might be interested.

The CHOICE study will test a program designed to improve the health of Veterans who drink alcohol. The study is sponsored by the National Institutes of Health.

Study overview:

- If you join this study, the study team will ask you questions about your health, mental health, and drinking. You will answer these questions by phone and in person. They will also ask you for a small amount of blood for lab tests.
- Half the Veterans in the study will be offered the extra services described in the enclosed information sheet. The goal of the study is to test whether these services are helpful.
- Agreeing to be in the study does not mean you have to try any extra services the study team may offer you.
- Agreeing to be in the study is completely voluntary and will not impact your existing care at the VA in any way.

What happens next:

- Please read the enclosed information sheet, and call the study's toll free number if you have questions: 1-800-781-5871. Otherwise, the study staff **will call you in about two weeks** to find out if you are interested in participating**.**
- If you do not want the study to contact you, please return the enclosed postage-paid postcard. You may also call them at 1-800-781-5871.
- The study team has enclosed $2 as a thank you for your time. If you would like to confirm that this study invitation is valid, you may call the VA's Institutional Review Board at 206-277-1715.

Sincerely, Dr. XYZ

Research Study Information Sheet

What is the purpose of this study?

- The study will test a program designed to improve the health of people who drink alcohol. We hope the results will help us provide better care for Veterans who drink.

How do I enroll in the CHOICE study?

- We will call you in about 2 weeks to tell you more about the study and answer your questions. We will ask you a few questions to see if you are eligible to participate.
- If you are interested and eligible, we will ask you to come to a VA Puget Sound medical center for an interview with a study team member. The visit will include a short survey to make sure you are still eligible for the study. You will receive $10 for coming to the visit and completing the short survey.
- If you are still eligible, the visit will last a total of about 2 hours. It will include a blood draw for lab tests and an in-depth survey about your health. Some of the questions will ask about drinking and depression. You will receive another $10 for finishing the interview.

What else does the study involve?

- We will ask you to do two surveys by phone. The first survey will last about 30 minutes and will happen 3 months after you enroll in the study. The second survey will last about 45 minutes and will happen 12 months after you enroll. To thank you for your help, we will give you $10 for doing the first survey and $15 for doing the second.
- We will ask you to do one blood draw at a VA facility 12 months after you enroll. You will receive $15 as a thank you for doing the blood draw.
- We will also offer half the Veterans in this study extra services to help manage health conditions that may be affected by drinking.

What are the extra services the study is testing that are offered for one year?

- The extra services include phone calls or in-person visits with a nurse, who will check the results of your blood tests for effects of alcohol use on your health. The nurse may also suggest medications that can help people drink less.
- This study is testing whether these services are helpful. If we offer you these extra services, it is your choice whether or not to accept them.

What else do I need to know?

- Choosing to be in this study or not will not affect your current health care.
- Being in this study is voluntary and you can stop at any time.
- You may call the study at 1-800-781-5871 if you have any questions.
- If you do not want to be in this study, please mail back the enclosed postcard. You can also call us at 1-800-781-5871, and we will not contact you again.

Telephone Script to conduct Brief Telephone Screen

* This script would be used at least 14 days after a potentially eligible subject has been mailed a recruitment letter, if they do not opt out of the study and for patients who self-refer to the Choice study by contacting the study team to be screened.

Introduction for proactive recruitment calls (not self-referrals)
May I please speak with Mr./Ms ______________ ? This is ___________________ calling from the VA.

Mr./Ms. ___________ , my name is ____________ . I am calling from the VA about a research study called the CHOICE Study. You should have received a letter about this study in the mail recently. You are being contacted because you are a primary care patient at a VA Puget Sound clinic and we thought that you might be interested in this study. Do you have a moment so that I may give you a little information about the study?

(Patient response)

IF PATIENT UNABLE TO TALK AT THAT TIME, BUT WILLING TO ANOTHER TIME, SCHEDULE A DATE AND TIME TO TALK WITHIN THE WEEK.

IF PATIENT ANSWERS “NO,” THANK PATIENT FOR HIS OR HER TIME. IF PATIENT ANSWERS “YES,” CONTINUE.

(Start here for self-referrals)
We are looking for volunteers to participate in a research study that is testing a program in the VA primary care clinics designed to improve the health of veterans who drink alcohol. We would like to ask some basic screening questions about you and about your drinking patterns. These questions should take about 3 minutes to complete. The phone screening is voluntary and you can stop answering questions at any time. You may consider some of this information to be personally sensitive. If you do not wish to provide this information over the phone, we would be happy to set up an in-person appointment instead.

Any information you provide will be kept strictly confidential and will be used only for research purposes. If it looks like you might be eligible for the study, I will explain more about the study, and, if you think you might be interested in participating, we will invite you to attend an in-person appointment at the Seattle or American Lake Division per your preference.
Would you be willing to answer some questions for our phone screen?

(Pause for patient response)

IF PATIENT ANSWERS “NO,” THANK PATIENT FOR HIS OR HER TIME. IF PATIENT ANSWERS “YES,” CONTINUE.

GENERAL INFORMATION

1. **Do you plan on receiving care from a VA Puget Sound facility for the next year?**
   ______ (0) No → NOT ELIGIBLE
   ______ (1) Yes

2. **(FEMALE ONLY) Are you currently pregnant or planning on becoming pregnant in the next 12 months?**
   ______ Yes, currently pregnant → NOT ELIGIBLE
   ______ Yes, planning on becoming pregnant → NOT ELIGIBLE
   ______ No

3. **(ADD FOR SELF-REFERRALS ONLY) How old are you?**
   ______ (0) Under 21 or Over 75 → NOT ELIGIBLE
   ______ (1) 21-75 years old

4. **(ADD FOR SELF-REFERRALS ONLY) Are you a VA employee?**
   ______ (0) No
   ______ (1) Yes → NOT ELIGIBLE

ALCOHOL HISTORY

Now I am going to ask you some questions about your alcohol use.

5. **How often do you drink alcohol?**

   ____

6. **What is your preferred drink?**

   ____

7. **IF FEMALE, ASK:**
   How many times during the past four weeks have you had four (4) or more drinks in a single day? By one "drink" we mean one 12 oz bottle of beer or wine cooler, one 5 oz glass of wine, or one shot of hard liquor.

8. **IF MALE, ASK:**
   How many times during the past four weeks have you had five (5) or more drinks in a single day? By one "drink" we mean one 12 oz bottle of beer or wine cooler, one 5 oz glass of wine, or one shot of hard liquor.

[Directions to Interviewer: Confirm the number of days. "Just to confirm, ____ days in the last 28 days you have had four/five or more drinks in a single day." If needed, repeat "How many times during the past four weeks have you had four(4)/five(5) or more drinks in a single day?"

<u>If patient reports less than 8 days</u> ask, "What do you typically drink on the days you drink?" If needed to help assess number of drinks add the following questions:

- What kind of alcohol (beer, ice beer, malt beer, wine, fortified wine, liquor, liqueur)
- How many (e.g. 5 beers, 4 glasses of wine, 3 mixed drinks, etc)?
- Ounces or milliliters for each drink (e.g. 12, 16, 22, 24, 40 oz of beer, and/or shot, nip, pint, 1/5, quart, half gallon, gallon of hard alcohol)?
- In the last 28 days, how many days have you had your typical amount?
  Determine if respondent meets criteria based on typical drinking]

9. In the last 90 days, have you received any alcohol treatment (VA or non-VA) through a halfway house or residential facility, a substance abuse counselor or mental health professional, an out-patient or in-patient program, or are you prescribed medications that help you cut down or quit drinking? Do not include AA or other Twelve-Step meetings.

______ (0) No

______ (1) Yes → NOT ELIGIBLE

10. Have you ever been in treatment or attended AA meetings for an alcohol problem?

____ Yes, but not in the last year
____ Yes, during the last year
____ No

Thank you. That is the end of the screen.

**SCREEN POSITIVE:**	**SCREEN FAIL:**
□ Getting care at VA in the next year	□ Not getting care at VA in the next year
□ Not pregnant or planning to become pregnant	□ Currently pregnant or planning on pregnancy
□ No current (in last 90 days) alcohol treatment	□ Current (last 90 days) alcohol treatment (VA or non-VA, but NOT AA or 12-Step
□ **≥ 8 heavy drinking days** in past 4 weeks (≥5 drinks for men, ≥4 drinks for women)<br>OR<br>□ **≥ 4 heavy drinking days** in past 4 weeks (≥5 drinks for men, ≥4 drinks for women) WITH **prior alcohol treatment**	□ Does not meet heavy drinking: **< 8 heavy drinking days** in past 4 weeks (≥5 drinks for men, ≥4 drinks for women)<br>AND<br>□ **< 4 heavy drinking days** in past 4 weeks (≥5 drinks for men, ≥4 drinks for women) WITH **prior alcohol treatment**
□ Age 21-75	□ Not age 21-75
□ Not a VA Employee	□ VA Employee

IF SCREEN POSITIVE:
"Your screening answers suggest you may be eligible for the trial.

I would like to explain a little bit more about the CHOICE study. The purpose of the study is to improve the health of veterans who drink alcohol. As part of the study, half of the patients will receive care as usual from their VA primary care provider. The other half of patients will receive care as usual from their VA primary care provider and, in addition, will be offered **optional** extra support to manage health conditions that may be affected by drinking. This includes in-person or telephone follow-up meetings, special labs to test for physical harm due to drinking, and medications that help patients decrease drinking if they choose. This study is testing whether these extra services will benefit patients. This extra care is completely optional and agreeing to participate in the study does not mean that you have to stop or decrease drinking or accept any extra services if they are offered to you.

If you are interested, I would like to schedule an in-person meeting with you at *(specify VA site)* to give you more information about the study and your potential role in it. During this meeting, if you are still interested in the study, I will also ask you to answer additional survey questions and ask you to go to the lab to give a small amount of blood for lab tests. The appointment may take up to 2 hours, so please make sure you are able to stay for that amount of time on the day you come in. In addition, not everyone will be eligible to participate in the study. For your time and effort, we will provide you $10 for coming in to the appointment and completing a few more screening questions. If you are eligible and complete all of the assessments, we will provide you with an additional $10.

Would you be willing to meet with me to learn more about the study?
(Patient Response)

IF YES (For self-referrals) – Great! Before we can schedule a time for you to meet with me, I will need to check with your primary care clinic to make sure you are eligible for the study. I can give you a call in the next few weeks to follow-up.

CONFIRM CONTACT INFORMATION AND LAST 4 DIGITS OF SSN (NAME, ADDRESS, SAFE PHONE NUMBER) OR ALTERNATE CONTACT INFORMATION BELOW.

Thank you for your time and your cooperation! If you have any further questions regarding the study please feel free to call me, _______, at our toll free number 1-800-781-5871. [End script here for self-referrals.]

IF YES – Great! Thank you very much. I can meet with you on the same day as your next appointment at the VA or whenever is most convenient for you. (If scheduling on the same day as their next health care appointment, ask if they know whether they will be getting any blood drawn. Confirm patient knows visit will be 2 hours and to please call to cancel or reschedule as early as possible, and provide enrollment coordinator's cell)

[SCHEDULE APPOINTMENT AND CONFIRM CONTACT INFORMATION (NAME, ADDRESS, SAFE PHONE NUMBER) IF THERE IS ALTERNATE CONTACT INFORMATION, RECORD BELOW.]

Name (and last 4 digits of SSN for self-referrals) ______________________________
Phone Number ______________________________
Address ______________________________

Name (if not the name of patient) ______________________________
Phone Number ______________________________
Address ______________________________

I am going to mail you an appointment reminder letter and our study's written informed consent document. Please feel free to read it over so you can learn more about the study. You can bring the consent form, along with any questions you may have, with you on the day of your appointment and I will answer any questions you have at your appointment. I will go over the informed consent document with you.

If you choose to take part in the study, we will be asking you for two additional contacts who would be able help us get in touch with you in case we are not able to reach you directly. It may be helpful to bring your cell phone and/or your address book

Thank you for your time and your cooperation! If you have any further questions regarding the study please feel free to call me, _______, at our toll free number 1-800-781-5871. I will call you to confirm our meeting the day before your appointment.

IF NO – Thank you, we really appreciate all your time today. We would still like to invite you to be interviewed one time, if you are willing. We are trying to learn more about alcohol related services that may be helpful for Veterans who are not interested in this study. The interview takes 45-60 minutes and we pay you $20 for your time and travel. Are you interested in participating?

IFYES: Schedule interview, Thank patient and end call.

IF NO: Thanks again for your time.

If you would like to know more about alcohol and your health, we would be happy to send you some information.

SCREEN FAIL: Thank you for your time. Your screening answers suggest you are currently not eligible for the study. However, if you would like to know more about alcohol and your health, we would be happy to send you some information.

Space to record what patient said and what needs to be done to follow-up (done by enrollment coordinator).

Department of Veterans Affairs	VA PUGET SOUND HEALTH CARE SYSTEM (663) RESEARCH CONSENT FORM	
SUBJECT NAME		SSN:
TITLE OF STUDY	**Considering Healthier Drinking Options in Collaborative Care**	
PRINCIPAL INVESTIGATOR	**Katharine A. Bradley, MD, MPH**	

LAY TITLE: CHOICE

Researchers:

Katharine Bradley, MD, MPH	Principal Investigator	(206) 287-2151
Daniel Kivlahan, PhD	Co-Investigator	(206) 764-2608
Andrew Saxon, MD	Co-Investigator	(206) 277-3770
Diane Greenberg, PhD	Co-Investigator	(206) 764-2965
Traci Takahashi, MD, MPH	Co-Investigator	(206) 277-5063
Evette Ludman, PhD	Co-Investigator	(206) 287-2917
Rachel Thomas, MPH	Study Coordinator	(206) 277-4161
Selin Caka, MA	Enrollment Coordinator	(206) 764-2068
Amy Lee, MPH	Enrollment Coordinator	(206)-764-2068
Gwen Lapham, PhD, MPH, MSW	Enrollment Coordinator	(206) 277-4583
Erika Holden, BA	Enrollment Coordinator	(206) 764-2068
Julie LaGuire, RN	Nurse	(206) 764-2412
Susan Ruedebusch, RN	Nurse	(206) 764-2068
Carol Achtmeyer, ARNP, MN	Nurse Practitioner	(206) 764-2932

24-hour emergency contact: Please call the CHOICE pager at (206) 416-1722 and enter in your 10-digit phone number after the beep.

You are invited to be in a research study. The purpose of this Consent Form is to give you the information you will need to help you decide whether to be in the study. Please read this form carefully. You may ask questions about the purpose of the research, what we would ask you to do, the possible risks and benefits, your rights as a volunteer, and anything else about the research or this form that is not clear. When we have answered all your questions, you can decide whether you want to be in the study. You are free to discuss this with friends or family. This process is called "informed consent."

1. **Purpose of research study and how long it will last:** The CHOICE Study is funded by the National Institutes of Health (NIH). This research study will test a new program to improve the delivery of effective medical care for Veterans who drink alcohol. We expect to enroll about 400 patients from the Veterans Affairs Puget Sound Health Care System (VAPSHCS).

SUBJECT'S IDENTIFICATION **(I.D. plate or give name-last, first, middle)**

IRB APPROVED
DEC 11 2013
VA PUGET SOUND
HEALTH CARE SYSTEM

STUDY TITLE: Considering Healthier Drinking Options in Collaborative Care

Why is the study being done? This study is for VA patients who drink alcohol. We know that alcohol affects many health conditions and can interact with medications. The purpose of the study is to test a program designed to improve the health of Veterans who drink alcohol.

How are you eligible? We are inviting you to participate if you were recently seen at a VA primary care clinic and reported drinking at levels that can affect your health. You must be 75 years of age or younger to be eligible.

2. Description of the study including procedures to be used: If you wish to join the study and sign this Consent Form, we will ask you to complete a series of surveys. We will ask you to complete an in-person baseline appointment, two telephone interviews, and two blood draws over a year. We will also be checking the long-term effects of the study through reviews of medical records.

Some patients will be offered support services that this study is testing to address any health problems that may be affected by alcohol. Research appointments and all blood draws will be at a VAPSHCS facility.

The CHOICE Support Program ***(for patients who by chance are offered extra care)***

This research will compare different types of patient care. If you choose to take part, you will be assigned by chance to one of two groups – Group A or Group B. As with the flip of a coin, you will have a 50/50 chance of being in either group.

If you are assigned to **Group A**:

- You will continue to receive regular care from your VA primary care provider.

If you are assigned to **Group B**:

- You will continue to receive regular care from your VA primary care provider; and
- You will also be offered extra services as part of the support program we are testing in this study to see if it will benefit patients. However, you do not have to accept any of the extra support that is offered to you.
- The services you would receive if you are in Group B will depend on *your preferences* and your personal health conditions.

IRB APPROVED
DEC 1 1 2013
VA PUGET SOUND
HEALTH CARE SYSTEM

Department of Veterans Affairs

VA PUGET SOUND HEALTH CARE SYSTEM (663)
RESEARCH CONSENT FORM

STUDY TITLE: Considering Healthier Drinking Options in Collaborative Care

All Group B patients will be offered the following services:

- An initial appointment with a CHOICE nurse, in person or by phone depending on your preference.
- An initial appointment with a nurse practitioner (NP) who will review your medical records and go over laboratory tests done for the study. The NP would also discuss your health conditions that might be worsened by drinking or medications that might interact with alcohol.
- Up to 16 visits with the CHOICE nurse. These visits would typically be weekly for 1 month, every 2 weeks for 1 month, and monthly thereafter for the year of the study, but the exact frequency would depend on your preference. Each nurse visit would be between 5 and 30 minutes.
- The 16 visits with the CHOICE nurse could include any of the following:
    - Review and monitoring of abnormal lab tests that can indicate harm due to drinking.
    - Review and monitoring of the effect of alcohol on your health conditions.
    - Review and monitoring of alcohol's interactions with medications you take.
    - Education about how drinking may affect your health conditions.
    - Helping you to set personal goals and planning activities to help you manage your physical health conditions and decrease your drinking, if you choose.
    - The study nurses will coordinate care with your VA primary care provider.
- Some Group B patients will be offered optional medications to help decrease drinking. If you were offered medications, the NP will discuss three medication options with you that are approved by the U.S. Food and Drug Administration *(naltrexone, disulfiram,* and *acamprosate).*
    - If a medication is recommended for you and you wish to try it, you will receive detailed information about the possible benefits and risks of that particular medication at the time it is prescribed.
    - If you choose to take one of these medications, you will need to schedule meetings with the NP to start or adjust medications.

IRB APPROVED
DEC 11 2013
VA PUGET SOUND
HEALTH CARE SYSTEM

Department of Veterans Affairs

VA PUGET SOUND HEALTH CARE SYSTEM (663)
RESEARCH CONSENT FORM

STUDY TITLE: Considering Healthier Drinking Options in Collaborative Care

We estimate that being in the support program will typically take 6 to 12 hours over the next year. At the end of the 1-year period, the support program will end. If this study is stopped early, these services will no longer be provided.

If you are in Group B, digital audio-recordings may be made of your nurse visits. Recordings will be used to verify the nurse visits are following the study protocol and to advise the nurses. Supervisors will review the audio-files to rate and provide feedback to the study nurses. Only approved research staff will have access to the computer audio-files.

The following are research procedures that both Groups A and B will complete:

In-Person Research Appointment

You recently completed a telephone interview. You were asked questions about your alcohol use. The next step in the enrollment process is your in-person interview with the enrollment coordinator. If you agree to take part, the coordinator will ask you to complete the following *at that appointment:*

- A brief screen to determine if you are eligible for the study. You will be asked questions that are similar to ones that you have already answered over the phone.
  Time estimate: 5 minutes

  Example question: *We will ask you to remember three objects and ask you what they are a few minutes later.*

- A written survey that asks about your drinking patterns, depression, post-traumatic stress disorder, and prior alcohol treatment.
  Time estimate: 20 minutes

  Example question: *"How often during the last year have you had a feeling of guilt or remorse after drinking?"*

- An in-person interview with the enrollment coordinator. Questions will be about your health, how you feel, family history of alcohol use, past treatment for alcohol use, and other information such as your education and race.
  Time estimate: 30 minutes

  Example question: *"Have you felt sad, low, or depressed most of the time for the last 2 years?"*

IRB APPROVED
DEC 11 2013
VA PUGET SOUND
HEALTH CARE SYSTEM

Medical Records

If you are by chance offered to receive services from the support program, CHOICE nurses and the nurse practitioner will document all clinical care in your VA medical records.

To understand the effects of the program we are testing, we will need to collect health care information. This information will be from your VA electronic medical records and from your Washington State health records. We would like to see your lab test results, which VA services you use, the kinds of medications you take, health care costs, and any hospitalizations inside or outside the VA. If you do not allow us to see your medical records, you will not be able to join this study. If you agree to be in this study, we will collect this information from your electronic medical records for 2 years before today's date and 11 years after today's date.

Telephone Interviews

The Group Health Research Institute (GHRI) Survey Research Program will conduct all telephone interviews. An interviewer from GHRI will call you for two phone calls:

- The first phone call will happen 3 months after your in-person research appointment.
  Time estimate: 20 minutes

- The second phone call will be 12 months after your in-person research appointment.
  Time estimate: 45 minutes

  Example question: *"How often during the last year have you had a feeling of guilt or remorse after drinking?"*

Lab Visits

During your in-person research appointment, the enrollment coordinator will walk you to the lab for a blood draw. VA lab personnel will draw blood (1-2 tablespoons) from a vein for three lab tests. These tests will give us more information about how alcohol affects your body.

Two of these tests are part of routine care at VAPSHCS. The results will be included in your medical records and made available to your VA provider. One test is not used at VAPSHCS. It will be analyzed at the Clinical Neurobiology Laboratory at the Medical University of South Carolina. The results of this lab test will only be used for research purposes. However, if you are offered extra support and choose to keep track of these labs, they will all be included in your medical records and made available to your VA provider.

IRB APPROVED
DEC 11 2013
VA PUGET SOUND
HEALTH CARE SYSTEM

Department of Veterans Affairs

VA PUGET SOUND HEALTH CARE SYSTEM (663)
RESEARCH CONSENT FORM

STUDY TITLE: Considering Healthier Drinking Options in Collaborative Care

After 12 months, you will need to give us another blood sample (1-2 tablespoons) for the same three lab tests. As before, two lab results will be included in your VA electronic medical records. The third will be sent to the lab in South Carolina to be used for research.

Each blood draw will take about 15 minutes to complete.

Summary of Research Activities	Baseline Appointment (or soon after)	In 3 Months	In 12 Months
Written survey	X		
In-person interview	X		
Telephone interview		X	X
Blood draw	X		X

3. Description of any procedures that may result in discomfort or inconvenience: You may be inconvenienced during the study when you are called to complete telephone surveys.

You may be asked questions of a sensitive nature. You may experience discomfort or distress while answering questions about your alcohol use. However, these questions are similar to the questions asked as part of routine clinical care and ones you have already been asked during your pre-screening telephone call. You are free to skip any questions that you don't want to answer.

You may become tired while answering the survey questions. We encourage you to take a break if you become too tired. You can schedule a time to finish the survey on another day.

Having your blood drawn can be uncomfortable and can sometimes cause a bruise. You may become faint or dizzy when blood is drawn. It doesn't happen often, but the puncture site can become infected. Only trained VA lab personnel will draw your blood. If your regular provider has ordered lab tests, the study tests can be drawn at the same time.

4. Potential risks of the study: There is a risk of loss of privacy (confidentiality). We have extensive measures in place to keep this from happening and expect these measures to protect your personal information.

If you are assigned to Group B, the CHOICE nurse will work with you to determine if your health might be harmed by alcohol. You will have the option of telephone calls with the nurse to discuss your health, lab tests to check for harm due to drinking, and prescribed medications to decrease your drinking. We cannot guarantee that support from the CHOICE nurse or medications from the nurse practitioner will help to improve your health.

IRB APPROVED
DEC 11 2013
VA PUGET SOUND
HEALTH CARE SYSTEM

Department of Veterans Affairs

VA PUGET SOUND HEALTH CARE SYSTEM (663)
RESEARCH CONSENT FORM

STUDY TITLE: Considering Healthier Drinking Options in Collaborative Care

If you are offered medications through Group B and choose to take them, there are some known side effects that may occur if you take *disulfiram, naltrexone,* or *acamprosate.*

Common Side Effects:

Common side effects of *naltrexone* are symptoms of nausea, abdominal pain/cramps or vomiting, diarrhea or constipation, joint and muscle pain, anxiety, depression, and difficulty sleeping. In rare cases, naltrexone can damage your liver.

Common side effects of *disulfiram* are skin rashes or itching, metallic aftertaste, headache, or drowsiness. Serious side effects include liver damage, blurred vision and, in rare cases, seizures and bipolar disorder.

Common side effects of *acamprosate* include skin rashes or itching, mild diarrhea, nausea, headache, depression, dizziness, dry mouth, or insomnia.

Other Cautions:

- If you take any of these medications and are capable of becoming pregnant, you should be using an effective method of birth control. If you become pregnant, you should let the study nurse know immediately and stop taking these medications.
- You should not take *naltrexone* if you have acute hepatitis or liver failure.
- If you are taking opiates to treat chronic pain, you should not take *naltrexone* because it blocks the effects of these medications.
- If you use opiate medications or drugs (morphine, methadone, oxycodone, hydrocodone codeine, or heroin), you should stop using these drugs at least 7 days before starting *naltrexone*. You must carry a wallet card or wear a medical bracelet at all times to alert clinicians that you are taking naltrexone in the event of an emergency.
- If you drink alcohol when you take *disulfiram,* it can make you feel sick. The most common effects of an interaction with alcohol include vomiting, blurred vision, chest pain, drowsiness, headache, and tremors. It should not be taken while intoxicated.
- You should always inform your health care providers of the medications that you are currently taking so that possible interactions can be monitored.

As with all medications, there may also be unanticipated side effects. The nurse practitioner and CHOICE nurse will ask you about undesired side effects that you may have and try to minimize them. You would need to tell the study nurse and your regular provider immediately if you have a

IRB APPROVED
DEC 11 2013
VA PUGET SOUND
HEALTH CARE SYSTEM

reaction to these medications. If you are selected for Group B, you can decide with the nurse practitioner if medications to help reduce your drinking are right for you. If you are prescribed these medications, you may decide to stop taking them at any time.

There may also be risks involved in your usual care, which you are advised to discuss with your health care provider.

The procedures in this study may involve risks that are currently unknown. We may need to contact you if we learn of a new study risk, even if you have completed the study. You may be asked to sign a new (updated) informed Consent Form to document that this new information has been explained to you.

5. **Potential benefits of study:** The study goal is to learn more about the drinking patterns of Veterans over a period of time. We would like to see if a support program will bring about higher quality health care and improve health conditions of these Veterans.

Your health may improve by being in the study. However, we cannot guarantee this. You may not benefit directly by participating in this study. We hope that this research will benefit other Veterans by showing whether added primary care support can minimize health risks from alcohol in the future.

6. **Other treatment available:** Taking part in this study does not stop you or your primary care provider from trying other kinds of treatment. You may always seek care you and your provider desire, whether or not you are being offered any extra health services in this study.

Standard care is available to Veterans who drink alcohol and have health issues. Standard care includes brief alcohol counseling with referral to an addictions specialist when needed.

7. **Use of research results / Confidentiality:** The information obtained about you will be kept confidential. However, for purposes of this study, the following list of people or groups may know that you are in this study. They will have access to your records, which may include your medical records:

- Research team members
- Seattle Institute for Biomedical and Clinical Research (the nonprofit institute that works with the VA to conduct research)
- The GHRI Survey Research Program
- The National Institutes of Health (NIH), the study sponsor
- Federal agencies including, but not limited to, the Food and Drug Administration (FDA), the Office for Human Research Protection (OHRP), the VA Office of Research Oversight (ORO), and the VA Office of the Inspector General (OIG), Government Accountability Office (GAO)
- The VA committees that oversee research, including the Institutional Review Board that oversees the safety and ethics of VA studies

IRB APPROVED
DEC 11 2013
VA PUGET SOUND
HEALTH CARE SYSTEM

- The Group Health committees that oversee research, including the Group Health Institutional Review Board and supporting staff, will have access to your study records but not your medical records

The access to your records, including your medical records, could be either for study-related purposes or to make sure that your records meet all legal, compliance, and administrative requirements. The reviewers will protect your privacy.

Your privacy is important to us. Study data will be stored on a secure computer within a password-protected database. Only approved research staff may access it. We will not place your name on any research data. Instead, we will assign a code number whenever possible to identify your information, such as on your lab tests and questionnaires. We will keep the master list that links your name to your code number in a secure-access computer specifically for research data. The master list will be stored in password-protected folders and will only be accessed by the Data Manager.

Once this study is completed, we will not use the code linking you to your data for any additional research. The code linking you to your data will be held in a secure database until the VA receives authorization to destroy it in accordance with federal records regulations. It may be several years before the code linking you to your data is actually destroyed. All coded data will be stored on secured computers or in file cabinets in locked offices. This coded data will be kept indefinitely.

A portion of all blood samples will be sent to the Clinical Neurobiology Laboratory at the Medical University of South Carolina for lab work to conduct a lab test. Samples will be mailed using a secure method and will not include your name. A unique study identification number will be used to label all your samples. Your blood samples will not be used for additional research and will be destroyed after the lab tests are completed.

Your name, address, and telephone number will only be shared with researchers at the Group Health Research Institute (GHRI) Survey Research Program. Survey interviewers will be collecting sensitive data during telephone surveys. They will be contacting you to complete telephone surveys at baseline, 3 months, and 12 months. All telephone survey information will be stored on password-protected computers that are only accessible to authorized personnel. All data will be sent to the Data Manager at VA's Health Services Research and Development Service (HSR&D) through a secure method at the end of each quarter.

The audio-recordings of the nurse visits will be de-identified. The audio-files will be labeled with your unique study code number and will not contain your name, social security number, or other identifying information. The audio-files will be stored on a secured computer server protected by a VA firewall in password-protected folders accessible only to approved research staff. The digital recorders used to audio-record interviews will be kept with the research staff or stored securely at all times. Please note that your voice is technically identifiable according to HIPAA patient privacy

rules, so we will do everything possible to protect your voice identity. The nurse will review a "Consent for Use of Picture and/or Voice" with you and ask for your consent before your nurse visit is recorded.

We have a Certificate of Confidentiality from the National Institutes of Health. The researchers will use the Certificate to resist any demands, even by a court of law, for information that would identify you. You may still share information about yourself or your part in this research as you see fit.

Any of this information that is disclosed will not be protected by the Certificate of Confidentiality to:

- The study sponsor.
- State or federal public health authorities to whom certain contagious diseases (tuberculosis, HIV, anthrax, syphilis) are reported (if we observe such diseases in any subjects).
- Law enforcement authorities any information that suggests the occurrence of child abuse, elder abuse, or your intent to immediately and substantially harm yourself or others.
- Your VA medical record any specific information as described elsewhere in this form.
- The VA Research Administration Office database your name, fact of participation in this study (including the study name), and contact information for the researcher.
- The VA office that manages payment to subjects your name, social security number, address, and the name of this study.
- State, federal, and institutional offices involved in auditing or compliance of research, risk management, patient safety, and financial controls (which includes the Food and Drug Administration)

In addition, any study test results or information that is included in your VA medical record will not be covered by the Certificate of Confidentiality and may be released if requested by a lawful subpoena or other lawful and appropriate request for the information. Persons who have access to your medical record will have access to any research-related information or documents that are in your record.

In the future, researchers may write publications using the information collected from this research study. Any future publications will not include any identifying information about you without your approval in writing.

IRB APPROVED
DEC 11 2013
VA PUGET SOUND
HEALTH CARE SYSTEM

We will use the information that we collect for this study only for research purposes, not for profit. However, researchers may use this research information in the future for the development of new ways to diagnose or treat diseases. Neither you nor your family will gain financially from discoveries made using the information or specimens that you provide.

Medical Record

If you are a VA patient, you already have a VA medical record. If you are not a VA patient, we will create a VA medical record for you. The creation of a VA medical record for you for the purposes of this study does not entitle you to any services at the VA beyond those services to which you are otherwise entitled.

We will put information about you from this study into your medical record. All authorized users of the national VA medical records system can have access to your medical record. This may include health insurance companies who are being billed for medical costs. This record will be kept forever.

8. Special circumstances: The VA requires some Veterans to pay co-payments for medical care and services. You will still have to pay these co-payments as long as they are not related to this research study.

You may have time and travel expenses due to coming in for the enrollment interview, completing phone surveys, returning for lab tests, or for appointments with study nurses. We will make every effort to schedule the final lab test at a time that is most convenient for you and when you will be traveling to the VA for an appointment.

You will be reimbursed:

- $2 pre-incentives for your telephone assessments with GHRI.
- $10 for the in-person baseline appointment.
- $10 for your time after you complete the baseline assessments, which include the enrollment interview, written survey, and baseline lab test.
- $10 for completing the 20-minute telephone survey at 3 months.
- $15 after 12 months for completing the 45-minute telephone survey.
- $15 for the final lab test at 12 months.

IRB APPROVED
DEC 11 2013
VA PUGET SOUND
HEALTH CARE SYSTEM

Cash will be mailed to your permanent mailing address after you complete each telephone assessment. It typically takes between 2 and 4 weeks for you to receive the payment, but it could take longer.

9. **Withdrawal from the study:** You do not have to take part in this study. If you are in this study, you can withdraw at any time. You will not be penalized for your decision to not participate or withdraw nor will you lose your VA or other benefits if you decide to do so.

The study physician has the right to terminate your participation in this study if he or she feels that it is not in your best interest to continue in the study. This termination will not require your consent.

If you decide to withdraw, or if you are terminated from the study, a person from the study team will then need to meet with you to discuss the necessary steps that you may need to take to end your participation in the study.

10. **Questions or concerns related to the study:** The study researchers (listed below) *must* be contacted immediately if:

- You think you may have been harmed or injured as a direct result of this research; and/or
- You have any questions regarding your medical care issues.

**During business hours (8:00 a.m. – 4:30 p.m.)**	Call Dr. Diane Greenberg (or covering research investigator) at (206) 764-2965.
**After business hours (nights and weekends)**	Call the CHOICE pager at (206) 416-1722 and enter in your 10-digit phone number after the beep.

You may contact the Institutional Review Board (IRB) – VA Office at (206) 277-1715 if you:

- Would like to speak with a neutral party who is not involved with this study;
- Have questions, concerns, or complaints about the research;
- Would like to verify the validity of the study; or
- Have questions about your rights as a research subject.

An IRB is an independent body made up of medical, scientific, and non-scientific members, who ensures the protection of the rights, safety, and well-being of subjects involved in research.

11. **Research-related injury:** Medical treatment will be provided, if necessary, by the VA if you are injured by being in this study. You will not be charged for this treatment. Veterans who are injured because of being in this study may receive payment under Title 38, United States Code, Section 1151. Veterans or non-Veterans who are injured may receive payment under the Federal Tort Claims Act.

You do not waive any legal rights by signing this Consent Form.

IRB APPROVED
DEC 11 2013
VA PUGET SOUND
HEALTH CARE SYSTEM

Department of Veterans Affairs

VA PUGET SOUND HEALTH CARE SYSTEM (663)
RESEARCH CONSENT FORM

STUDY TITLE: Considering Healthier Drinking Options in Collaborative Care

12. Research subject's rights: I have read or have had read to me all of the above. The study has been explained to me, including a description of what the study is about and how and why it is being done. All of my questions have been answered. I have been told of the risks and/or discomforts, possible benefits of the study, and other choices of treatment available to me. My rights as a research subject have been explained to me and I voluntarily consent to participate in this study. I will receive a signed copy of this Consent Form.

I agree to participate in this research study as you have explained it in this document.

______________________________ ______________
Subject Signature **Date**

____
Print Name of Subject

______________________________ ______________
Signature of Person Obtaining Consent **Date**

____
Print Name of Person Obtaining Consent

IRB APPROVED
DEC 11 2013
VA PUGET SOUND
HEALTH CARE SYSTEM

SUPPLEMENT B: Fictional Example of Template for EHR Engagement Note

RN:						CHOICE study CCT Clinical Review Case							Next visit		
Subj ID	Initials	Age	Sex	Visit #	Visit Date	Enrl Date	Attending	Res	Med	Update			Notes		
5555	js	61	m	1	07/10/13	06/20/13	jones								
AUDIT	Date			PHQ9	Date	%CDT	Date	MCV	Date	GGT	Date	BP	Date	Pulse	Date
17	06/20/13			11	06/20/13	2.1	06/20/13	96.8	06/20/13	44	06/20/13	128/86	06/20/13	66	

Patient Goals: to enjoy grandson

Psychiatric:
ATC completed 1995
dx major depression
has MH counselor

Social:
divorced ; parents living; stays with Mom
currently seeking new AA group

Summary of Intervention:
reconnect with sober friends; try out AA again
be compliant with medications
interested in naltrexone

Readiness for change: contemplation
Motivators: grandchild
Facilitators: sober Friends ; family
Barriers: old friends that use alcohol; pain

Medications: discussed naltrexone

Plan: follow up with support and explore abstinence

SUPPLEMENT C: Fictional CHOICE Patient Roster

Choice Intervention Report

Studyid	Age, Sex	Rand	Last Visit	CDT (BL,MR) (0 - 1.6)	MCV (BL,MR) (81 - 98)	GGT (BL,MR) (0 - 50)	Audit (BL,MR)	PHQ (BL,MR)	Meds	Comment
**Nurse #1**										
4966	61, M	04/02/14	1, 05/13/14	1.3,--	99.7,--	30,--	15,--	3,--		appt MV 5/26
4317	32, M	03/31/14	2, 05/07/14	1.9,--	93.4,--	38,--	13,--	6,--		appt 6/4 MV
4631	45, F	03/25/14	1, 04/08/14	1.4,--	88.6,--	86,--	16,--	5,--		
4594	37, M	11/20/13	11, 05/04/14	0.8,--	94.4,--	11,--	26,--	6,4	N,03/06/14	appt 5/27
4183	25, M	10/30/13	3, 02/28/14	1.2,--	86.8,--	55,--	10,--	5,--	-	
3567	66, M	10/16/13	8, 05/22/14	2.6,--	94.3,--	21,--	17,--	10,--	-	
3476	40, F	10/09/13	5, 04/010/14	3.7,--	101,--	85,--	10,--	9,--	-	
2434	29, M	10/03/13	7, 05/19/14	1.3,--	100.4,96.6	15,12	18,12	6,2	-	
3561	70, M	09/22/13	2, 11/12/13	8.7,6.3	104.9,104.9	38,52	23,19	2,8	-	
**Nurse #2**									-	
4790	59, M	04/20/14	1, 05/21/14						-	
5031	65, M	04/07/14	4, 05/05/14	2.1,--	92.2,--	19,--	19,14	10,6	-	
4727	37, F	03/21/14	1, 04/30/14	3.3,--	100.2,--	22,--	19,10	5,--		
4430	65, M	03/14/14	3, 05/22/14	2.8,--	96.8,--	23,--	15,--	9,20	N,03/24/14	
4574	63, M	03/12/14	3, 05/10/14	2.8,--	101,--	887,--	17,--	2,--	-	
3271	49, F	02/27/14	5, 05/19/14	1.5,--	94.3,--	35,--	15,--	4,--	-	
4478	55, M	02/12/14	4, 04/30/14	1.2,--	93.4,--	15,--	25,18	13,23	-	
2825	67, M	01/22/14	2, 02/24/14	1.3,--	99.5,98.5	59,--	24,--	8,--	-	has N
4251	63, M	01/19/14	7, 05/10/14	4.6,--	100.3,93.3	56,68	11,11	11,14	-	f/u 5/22
4045	32, M	01/16/14	6, 05/06/14	2,--	98.6,--	12,--	25,4	16,21	A,04/104	acam str 4/18

SUPPLEMENT D: Comparison of Enrolled and Non-enrolled Samples

Among participants who were initially identified for possible inclusion in the CHOICE trial, characteristics among those who enrolled, were found to be ineligible, and who refused to participate

	Mean (SD)			P-value**	
**Characteristic**	**Enrolled**	**Ineligible**	**Declined or not reached**	**Ineligible vs. Enrolled**	**Declined/not reached vs. Enrolled**
Among all potential participants, including referrals	(n = 304)	(n = 1574)	(n = 2074)		
Age*	51.4 (±13.8)	47.8 (±15.0)	51.0 (±14.7)	0.0003	0.89
Age, among Males*	52.2 (±13.8)	48.7 (±15.1)	51.9 (±14.7)	0.001	0.71
Age, among Females	43.7 (±11.7)	42.8 (±13.2)	44.5 (±13.1)	0.68	0.75
Male, n (%)	275 (90.5%)	1,334 (84.8%)	1,820 (87.8%)	0.009	0.19
AUDIT-C				< 0.0001	< 0.0001
Mean (SD)	7.0 (±2.1)	6.4 (±2.0)	6.4 (±1.9)		
Missing, n (%)	20 (6.6%)	32 (2.0%)	14 (0.7%)		
AUDIT-C, among Males				< 0.0001	< 0.0001
Mean (SD)	7.2 (±2.1)	6.7 (±1.9)	6.6 (±1.8)		
Missing, n (%)	19 (6.9%)	31 (2.3%)	13 (0.7%)		
AUDIT-C, among Females				0.061	0.035
Mean (SD)	5.5 (±1.7)	4.9 (±1.6)	4.8 (±1.4)		
Missing, n (%)	1 (3.4%)	1 (0.4%)	1 (0.4%)		
Excluding referred patients	(n = 284)	(n = 1542)	(n = 2074)		
Age	51.1 (±14.2)	47.7 (±15.0)	51.0 (±14.7)	0.0008	0.76
Age, among Males	52.0 (±14.2)	48.6 (±15.2)	51.9 (±14.7)	0.002	0.7
Age, among Females	43.0 (±11.2)	42.9 (±13.2)	44.5 (±13.1)	0.9	0.56
Male, n (%)	256 (90.1%)	1,303 (84.5%)	1,807 (87.7%)	0.013	0.28
AUDIT-C	7.0 (±2.1)	6.4 (±2.0)	6.4 (±1.9)	< 0.0001	< 0.0001
AUDIT-C, among Males	7.2 (±2.1)	6.7 (±1.9)	6.6 (±1.8)	< 0.0001	< 0.0001
AUDIT-C, among Females	5.5 (±1.7)	4.9 (±1.6)	4.8 (±1.4)	0.061	0.035

*4 missing among ineligible participants
**P-values test for a difference in distribution across categories: for continuous variables, Wilcoxon rank sum test; for categorical variables, either Fisher's exact test (variables with 2-3 categories) or analysis of variance (variables with ≥4 categories).
